# Supplementary material for: An exploratory computational analysis in mice brain networks of widespread epileptic seizure onset locations along with potential strategies for effective intervention and propagation control
Source: Front Comput Neurosci. 2024 Feb 26;18:1360009. doi: 10.3389/fncom.2024.1360009 (PMC10925689; doi:10.3389/fncom.2024.1360009)
Supplement: Supplementary file 1 [file Data_Sheet_1.PDF]

Supplementary Material

1 ADDITIONAL STRUCTURAL CONNECTIVITY MATRICES GENERATED FROM THE ALLEN CONNECTOME

In **Figure** S1, we show in a logarithmic scale the SC of the 20 generated SC. We preserve the structural properties of the Allen connectome (see **Figure** 1 in the main article). In **Figure** S2, we display the difference between the original weight c_{ji} and the new weight c'_{ji} of their connections. Red elements indicate strengthened connections. Blue elements indicate weakened connections. While conserving the overall structure of the SC, there is a significant change in some of the strongest edges weight.

2 NETWORK EDGE REMOVAL

In **Figure** S3, we show two resection procedures for the localization of epileptic seizures initiating in l CA1. In **Figure** S3(A), we remove the inter-hemispheric pathway l CA1 $\nrightarrow$ r CA3 and the pathways leading from the EZ to extra-hippocampal left areas, namely left Entorhinal Cortex (l ENTl) and left Subiculum (l SUB). In **Figure** S3(B), we remove the inter-hemispheric pathway l CA1 $\nrightarrow$ r CA3 and the pathways leading from l ENTl, l SUB to high-centrality nodes of the brain network, namely the left Ectorhinal Cortex (l ECT), Perirhinal Cortex (l PRh) and Temporal Association (l TEa) areas. With these two procedures, we achieve seizure localization in a reduced number of brain areas, which suggests that network hubs are important for epileptic seizure propagation.

In **Figure** S4(A), we present the effects of outgoing weight reduction and edge removal approaches *Case I* (l CA1 $\nrightarrow$ r CA3 and l CA1 $\nrightarrow$ l DG), *Case II* (l CA1 $\nrightarrow$ l DG and r CA3 $\nrightarrow$ r CA1) and *Case III* (l CA1 $\nrightarrow$ l CA3) for the left-field CA1 area in the 20 generated randomization of the Allen connectome. In **Figure** S4(B), we present the effects of outgoing weight reduction and edge removal approaches *Case I'* (l DG $\nrightarrow$ r DG and l DG $\nrightarrow$ l CA1), *Case II'* (l DG $\nrightarrow$ l CA1 and r CA3 $\nrightarrow$ r DG) and *Case III'* (l DG $\nrightarrow$ l CA3) for the left Dentate Gyrus area in the 20 generated randomization of the Allen connectome. The two dashed lines show the upper (resp. lower) threshold of the largest outgoing weight separating the widespread from localized seizures (see **Figure** 10 and relevant discussion in the main manuscript for more details). When applying outgoing weight reduction, the progressive decrease of the EZ's outgoing connective strength leads its connectivity towards lower eigenvector centrality values, and especially lower strongest outgoing weight values. In all 20 generated SC matrices, seizure localization is achieved when the EZ passes below the ω_{lower} threshold. The approach *Case III* also results in reducing the EZ's strongest outgoing weight to value below ω_{lower} threshold. The edge removal approaches *Case I* and *II* (resp. *Case I'* and *Case II'* mildly also reduce the EZ's eigenvector centrality, and most of the time leads to seizure localization without any modification of its strongest outgoing connection.

3 FUNCTIONAL CONNECTIVITY BEFORE AND AFTER STRUCTURAL INTERVENTIONS

We also use functional analysis to evaluate time correlation between spatially distant brain areas' activity. To simulate functional connectivity, we compute 10 minute-long time-series of exclusively non-epileptogenic Epileptors coupled through the SC, to reproduce normal brain activity. The first 10 seconds of these time-series are removed to discard the initial bursting due to random initial conditions (the system

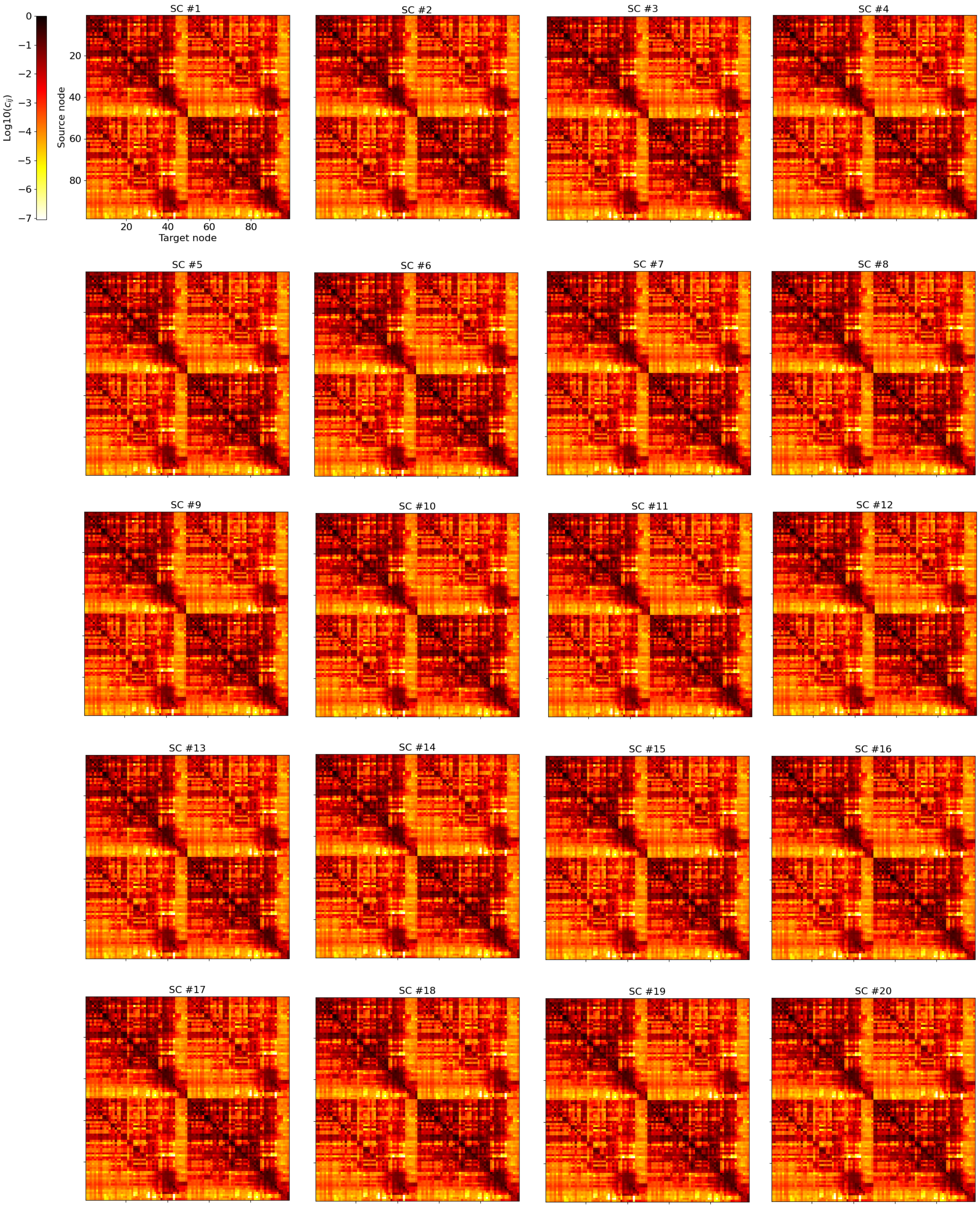

Figure S1. Allen SC matrices used for statistical analysis. For the 20 generated SC, we display the new weight matrix. We show the logarithm of the weight of each connection.

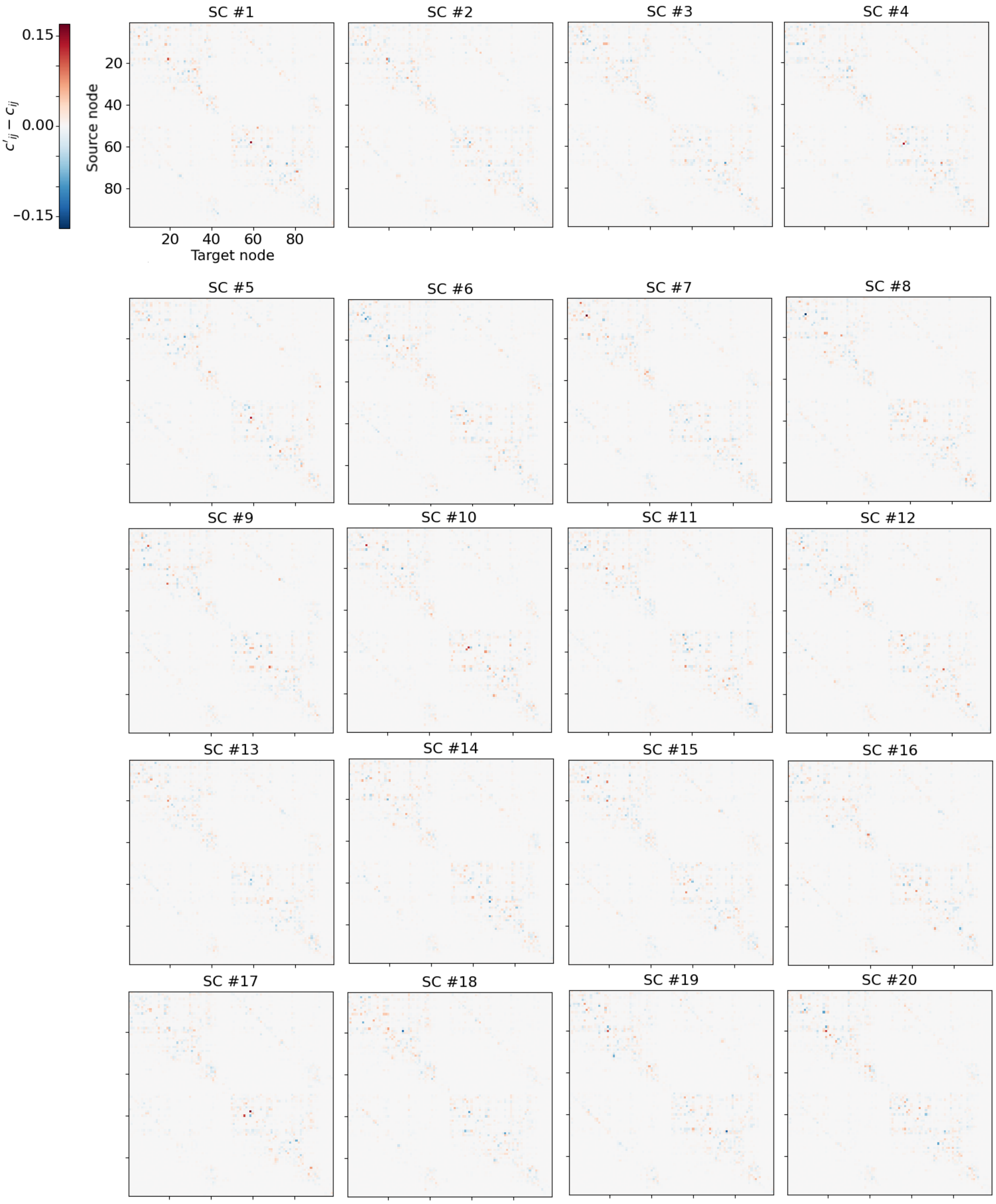

Figure S2. Weight value difference between the additional SC matrices and the original Allen connectome. For the 20 generated SC $SC' = (c'_{ji})$, we display the weight difference $c'_{ji} - c_{ji}$ between each respective matrix entry value.

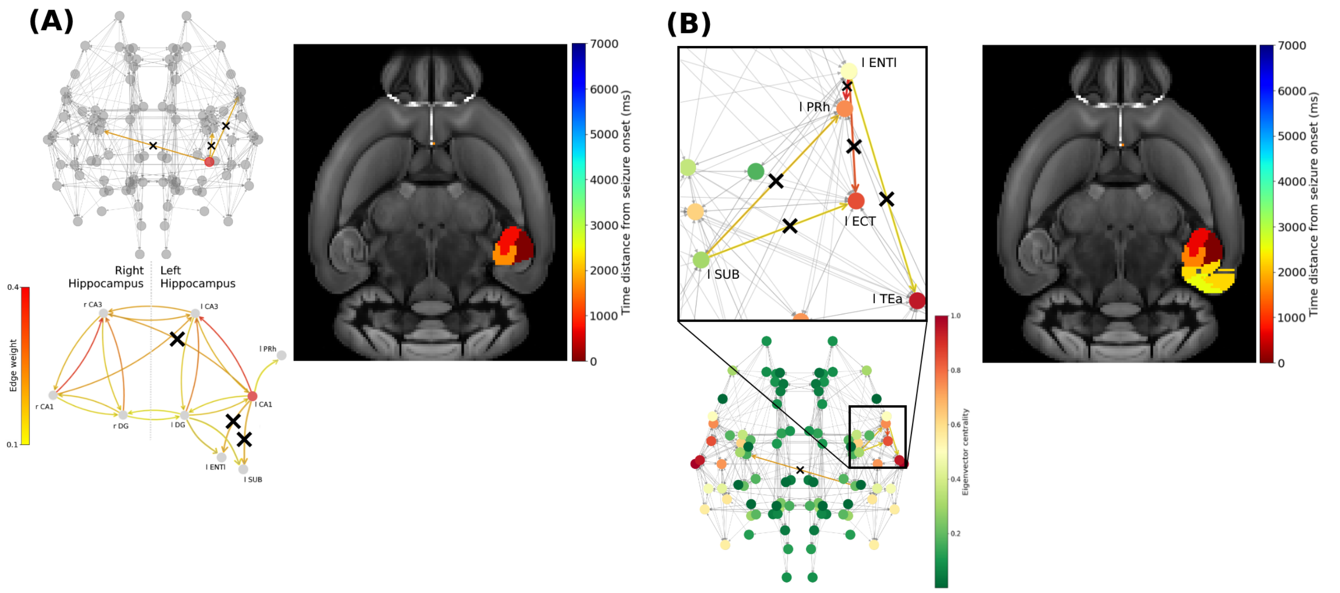

Figure S3. Widespread seizure prevention by edge removal in the left-field CA1.. The upper left panels show the brain network. Crosses indicate the removed connections, and the edges' color shows their respective weight. The right panels show the time distance between seizure initiation in the EZ and its onset in each brain area. **(A)** Seizure propagation in the Allen connectome after removing the inter-hemispheric strong connection l CA1 $\nrightarrow$ r CA3, and the two strong connections leading from the EZ to outside of left Hippocampus (namely the lateral part of the Entorhinal Cortex ENTl and the Subiculum (SUB)). The lower left panel illustrates the left extra-hippocampal connections and their weights values are given in the left colorbar. **(B)** Seizure propagation after removing the inter-hemispheric strong connection l CA1 $\nrightarrow$ r CA3, and all strong connections ($c_{ji} > 0.1$) leading from l ENTl, l SUB to networks hubs, namely the left Ectorhinal Cortex (ECT), Perirhinal Cortex (PRh) and Temporal Association (TEa) areas. The color of the nodes show their normalized eigenvector centrality.

requires some time to reach its stable state due to transient effects). The Epileptor activity is converted into simulated BOLD activity time-series using hemodynamic response functions implemented in TVB (Friston et al., 2000).

The Functional Connectivity (FC) matrix FC_{ij} is measured by the Pearson Correlation Coefficient r, i.e. by correlating simulated BOLD time series data between every pair of nodes in our network graph (Stephan and Friston, 2009; Ladwig et al., 2022):

$$r_{ij} = r(x_i, x_j). \tag{S1}$$

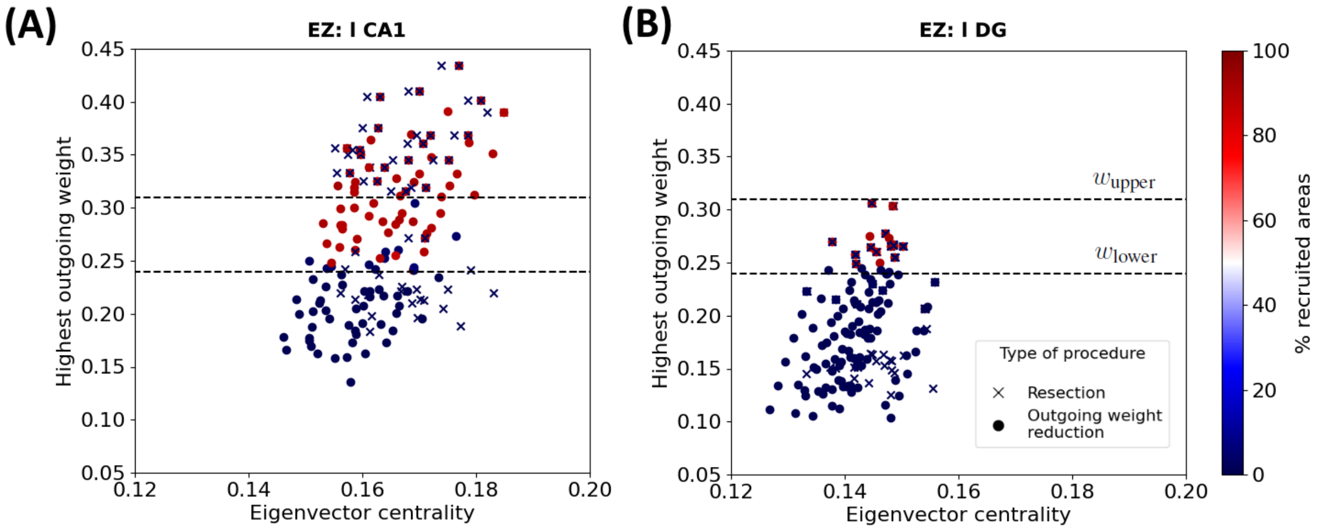

Figure S4. EZ connectivity and percentage of seizure-recruited areas after outgoing weight reduction and edge resection in the 20 generated connectomes. Each point shows the eigenvector centrality and the largest outgoing weight value in one of the 20 randomized connectomes of **(A)** left-field CA1 and **(B)** left Dentate Gyrus, before and after applying outgoing weight reduction (circles) and edge removal strategies presented in **Figure** (6) and **Figure** (7) (crosses). The color of each point shows the percentage of recruited brain areas when the seizure starts in the corresponding node. The two dashed lines show the upper (resp. lower) threshold of the largest outgoing weight from separating the widespread from localized seizures.

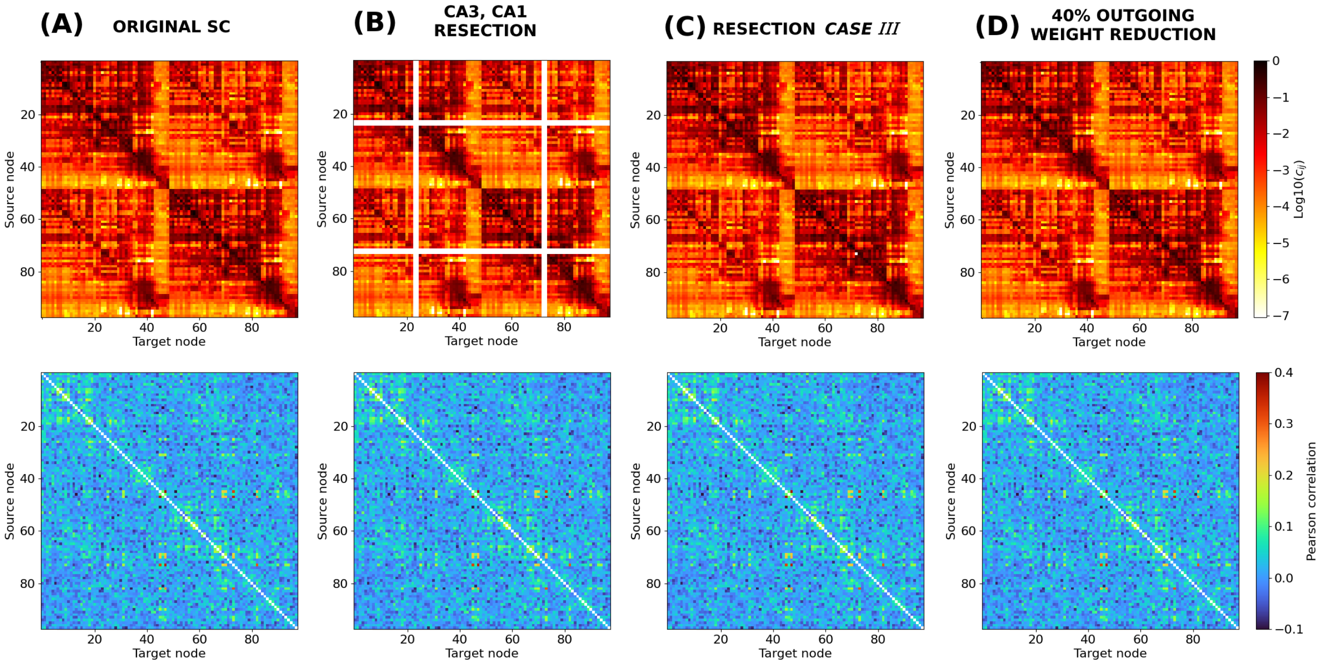

Figure S5. Connectome structure and functionality after resection and outgoing weight reduction procedures. We show the network structural connectivity (top) and functional connectivity (bottom) matrices. **(A)** Original connectome, **(B)** after the resection of left and right CA1 and CA3 areas, following the procedure presented in (Melozzi et al., 2017), **(C)** after applying resection *Case III* and **(D)** after applying l CA1 outgoing weight reduction of 40%.

In **Figure** S5, we show the original Allen SC matrix (panel (A)) together with modified ones, following effective seizure localization strategies. Panel (B) shows the SC after applying a standard resection technique presented in Melozzi et al. (2017). This approach consists in the removal of the CA1 and CA3 areas in both hemispheres. Panel (C) shows the SC after applying resection *Case I*, e.g. the resection of a single connection l CA1 $\nrightarrow$ l CA3, and panel (D) shows the SC after applying a 40% outgoing weight reduction on l CA1. With the latter two options, we minimize the structural modifications compared to the CA1, CA3 resection procedure Melozzi et al. (2017). By setting our system at a "healthy" (i.e., modeling all network nodes as non EZ), we do not manage to observe notable difference between the FC produced by the original SC matrix and the FC matrices produced with the different intervention procedures introduced in our manuscript. Here we show only a few typical examples. Note that we have also attempted to estimate matrix distances, e.g. by calculating the Pearson correlation coefficient between different pairs of matrices (see e.g. (Popovych et al., 2021; Manos et al., 2023)), but we did not observe striking dissimilarities (results not shown here). It is also worth noting that at this stage we did not aim to compare our simulated time series (or FC matrices) to experimental neuroimaging mice signals.

REFERENCES

Friston, K., Mechelli, A., Turner, R., and Price, C. (2000). Nonlinear responses in fmri: The balloon model, volterra kernels, and other hemodynamics. *NeuroImage* 12, 466–477. doi:https://doi.org/10.1006/nimg.2000.0630

Ladwig, Z., Seitzman, B. A., Dworetsky, A., Yu, Y., Adeyemo, B., Smith, D. M., et al. (2022). Bold cofluctuation 'events' are predicted from static functional connectivity. *NeuroImage* 260, 119476. doi:10.1016/j.neuroimage.2022.119476

Manos, T., Diaz-Pier, S., Fortel, I., Driscoll, I., Zhan, L., and Leow, A. (2023). Enhanced simulations of whole-brain dynamics using hybrid resting-state structural connectomes. *Front. Comput. Neurosci.* 17, 1295395. doi:10.3389/fncom.2023.1295395

Melozzi, F., Woodman, M., Jirsa, V., and Bernard, C. (2017). The virtual mouse brain: A computational neuroinformatics platform to study whole mouse brain dynamics. *eNeuro* doi:10.1101/123406

Popovych, O. V., Jung, K., Manos, T., Diaz-Pier, S., Hoffstaedter, F., Schreiber, J., et al. (2021). Inter-subject and inter-parcellation variability of resting-state whole-brain dynamical modeling. *NeuroImage* 236, 118201. doi:10.1016/j.neuroimage.2021.118201

Stephan, K. and Friston, K. (2009). Functional connectivity. *Encyclopedia of Neuroscience* , 391–397doi:10.1016/b978-008045046-9.00308-9
